# Delayed Surgical Treatment of a CE1 Lung Cyst Resulting in Pericystectomy of CE4 Cyst

**DOI:** 10.1155/2024/5167805

**Published:** 2024-03-14

**Authors:** Gian Luca D'Alessandro, Agostina Pontarelli, Armanda Leka, Dino Casazza, Raffaella Lissandrin, Tommaso Manciulli, Annarita Botta, Roberto Parrella, Enrico Brunetti, Pietro Rinaldi

**Affiliations:** ^1^Department of Clinical Surgical Diagnostic and Pediatric Sciences, University of Pavia, Pavia, Italy; ^2^Unit of Respiratory Infectious Diseases, Cotugno Hospital, Azienda Ospedaliera dei Colli, Naples, Italy; ^3^Unit of Thoracic Surgery, Monaldi Hospital, Azienda Ospedaliera dei Colli, Naples, Italy; ^4^San Matteo Hospital Foundation, Pavia, Italy; ^5^Department of Clinical and Experimental Medicine, University of Florence, Florence, Italy

## Abstract

Lung is the second most common locationof cystic echinococcosis (CE), after the liver. Diagnosis of lung CE is often incidental, and clinical manifestations depend on the location and size of the cyst, the most common being chest pain, shortness of breath, expectoration of fragments of endocyst, and haemoptysis. Surgery is the primary treatment, with a minor role for medical therapy. Delayed diagnosis and treatment may have important consequences. We present a case of lung CE in whichsurgical treatment was delayed due to the first wave of COVID-19. Since surgery could not be performed immediately, the patient was kept on albendazole and the cyst stage moved from CE1 to CE3a, to CE4, eventually requiring a more aggressive pericystectomy instead of the commonly performed endocystectomy. The clinical and imaging characteristics of a rare CE4 cyst of the lung are reported.

## 1. Introduction

Cystic echinococcosis (CE) is a zoonosis caused by the larval stage of the cestode *Echinococcosis granulosus s.l.*. It mainly affects patients from poor areas where sheep raising is practiced. Cysts may form in any internal organ, but the liver and lungs are the main locations[1]. Cyst rupture and dissemination (either through body cavities or via hematogenous spread) can result in secondary CE [1]. Diagnosis of CE is based on imaging, while serology has an ancillary role [2]. Cysts are staged using the “WHO Informal Working Group on Echinococcosis” classification, which also directs subsequent management of uncomplicated cases [3]. Lung lesions can be diagnosed by X-ray in most patients with a compatible history, while CT is used for pre-surgical evaluation [4]. Lung cysts can be treated with albendazole (ABZ) if they are small and uncomplicated [4], whereas cysts larger than 5 cm and smaller complicated cysts should be managed surgically, sparing as much parenchyma as possible [5]. Uncomplicated lung cysts initially may be asymptomatic or present with cough, chest pain, and shortness of breath. Here, we report a case of lung echinococcal cyst in which the delay of surgical intervention due to disruption of surgical activity during the first COVID-19 wave resulted in a CE4 stage cyst, rarely seen in the lung, eventually treated with pericystectomy.

## 2. Case Presentation

**Figure 1:**
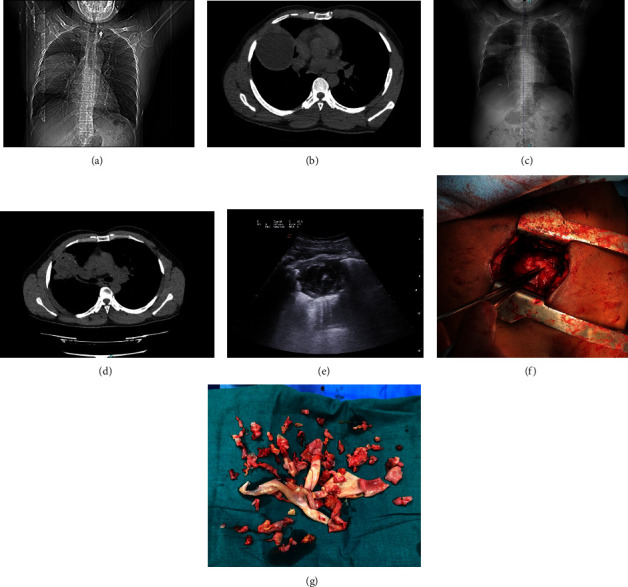
Radiological and surgical findings. (a, b) Thoracic imaging at the time of diagnosis showing a liquid cyst; (c, d) thoracic imaging right before surgery showing a decrease in cystic volume, air within the cyst, and pericystic thickening; (e) ultrasound of the lung cyst carried out during the hospitalization before surgery showing the complex US structure of the cyst, with the “ball of wool sign”; (f, g) intraoperative findings showing the thoracotomic approach and the shreds of pericyst requiring careful dissection.

**Figure 2:**
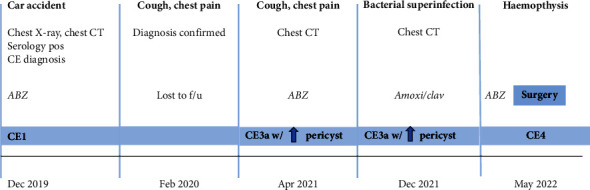
Timeline illustrating the events presented in our case report. ABZ = albendazole; CT = computer tomography; amoxi/clav = antibiotic treatment with amoxicillin/clavulanic acid.

A 36-year-old Ghanaian man, living in Italy since 2007, with a history of hypertension, was admitted to the E.D. of a tertiary hospital in the Campania region, Italy, in December 2019, following a car accident. A chest X-ray and CT scan documented an 11 × 7 cm cystic lesion localized in the right superior lobe of the lung without pleural effusion. The scan also showed a cystic lesion of the spleen. The patient was transferred to a tertiary care hospital in Naples, where imaging confirmed the lung cyst (Figures 1(a) and 1(b)) and serology for *E. granulosus*tested positive. Medical therapy was started with albendazole (ABZ) and the patient was discharged after being scheduled for surgery. However, he was lost to follow-up after a few weeks. In February 2020 he experienced sudden chest pain and expectoration of yellowish mucus and presented once again to the E.D. of the closest hospital. A CT scan of the chest ruled out life-threatening conditions, the diagnosis of lung CE was confirmed and the patient was discharged because surgery was limited due to COVID-19. The following year, in April 2021, another episode of expectoration of fragments of the endocysts and chest discomfort occurred, and the patient was admitted to the Pulmonology Unit of another hospital in Naples. ABZ was started once again, and a new CT scan of the chest showed a change in the appearance of the lung cyst, which was now surrounded by a thick wall with fluid content and wavy lines inside the cavity. A homolateral pleural effusion was seen, as well as mediastinal and right peribronchial lymphadenopathies, with a necrotic component. Indication for surgery was confirmed, but again surgery was not possible because of the ongoing COVID-19 pandemic. Radiological and clinical follow-up was carried outby a multidisciplinary team including infectious diseases specialists and thoracic surgeons at the Monaldi and Cotugno Hospitals in Naples. A CT scan of the chest performed in December 2021 showed a reduction in the size of the lung lesion, with the appearance of intracystic small air crescent and perilesional atelectasis. Bacterial superinfection of the cyst was suspected (leukocytosis, fever, and contrast-enhanced CT findings showing pericystic inflammation) and treated empirically with amoxicillin/clavulanate. The patient was referred to the WHO-Collaborating Centre for the Clinical Management of Cystic Echinococcosis in Pavia and was rescheduled for surgery but developed haemoptysis in May 2022, which lead to emergency surgery. The preoperative CT scan confirmed the above-described changes in the radiological appearance of the cyst (Figures 1(c)–1(e)), and the bronchoscopic examination ruled out major cysto-bronchial fistulisations. Surgical pericystectomy with a thoracoscopic-thoracotomic hybrid approach with bronchial saturation was performed after positioning of hypertonic-soaked gauze pads and subsequent lavage with hydrogen peroxide and povidone-iodine of the pleural cavity was carried out (Figures 1(f) and 1(g)). The patient recovered and continued therapy with ABZ, suspended in October 2022 after a 6-month course, when US examination of the abdomen showed almost complete solidification of the splenic cyst. The postoperative course was free of complications, and the patient is currently well and asymptomatic. A timeline of the events is presented in Figure 2.

## 3. Discussion

CE cysts in humans develop mainly in the liver (70% of cases) and lungs (20%). Cysts in the lungs tend to grow faster than in the liver [4]. Although temporarily silent, all lung cysts carry the risk of perforation [6]. Local complications of pulmonary CE include cyst rupture, superinfection, and mass effect of the cyst [7]. The cyst can form cysto-bronchial and cysto-pleural fistulae. The former causes expectoration of cyst fluid and portions of the endocyst and haemoptysis. Fistulized cysts are prone to superinfection with chronic lung abscess formation. Different treatment options are available for liver cysts, where a stage-specific approach has been recommended (medical therapy, surgery, percutaneous treatment, and watch and wait approach) [4]. However, the anatomical structure of the lung makes surgery the only option for large cysts. Benzimidazoles may be used in small cysts, as stated in the WHO-IWGE recommendations [4, 8], although failure rates of around 30% have been reported [8]. Percutaneous treatments are not indicated in lung CE patients, due to the high risk of rupture and dissemination and the same risk is present for larger cysts treated with benzimidazoles [9]. Hence, in lung CE, surgery must be employed for active and transitional cysts larger than 5 cm or for complicated cysts of any stage [4, 10, 11]. Prompt surgical treatment or treatment with ABZ in selected cases allow cure in the majority of cases, with relatively few recurrences and—in expert hands—minimal complications [10, 12, 13]. While not indicated as treatment in large pulmonary cysts due to the risk of rupture [14], all surgical interventions on the cyst require the use of a one-monthperioperative prophylaxis with ABZ (beginning from the day before the procedure), to avoid surgery-related CE dissemination [15].

The impact of the COVID-19 outbreak on healthcare systems, with the almost complete interruption of surgical activities, prevented our patients from being treated early [16]. The radiological findings in May 2022 (volume reduction, intracystic air bubbles, and pericystic consolidation) correlate with rupture and infection of the lung CE cyst [5, 7]. Complicated cysts in the lung are associated with higher postoperative morbidity and mortality [6]. The elapsed time probably allowed for the formation of an inflammatory tissue around the lung cyst, increasing the thicknessof the pericyst. The CT scan and ultrasound of the lung cyst performed immediately before surgery showed solidification of the content (CE4) and a thick cystic wall, a pattern rarely observed in lung CE (Figure 1(e)). The patient was initially treated with ABZ, then he developed a bronchial fistula that led episodes of expectoration of parasitic material. Chronic inflammation caused the thickening of the pericyst , assuggested by the presence of increased hilar lymph nodes. Bacterial superinfection (neutrophilia, increase in C-reactive protein) was noted. Since pericystic inflammation is known to occur following the administration of ABZ [17], it might have had a role in blood vessel erosion triggering haemoptysis. Chronic inflammation and the increased thickness of the pericyst may also have prevented the occurence ofmajor bronchial fistulas that were absent at the preoperative bronchoscopic examination. [10]. [14], [10].

**Figure 3:**
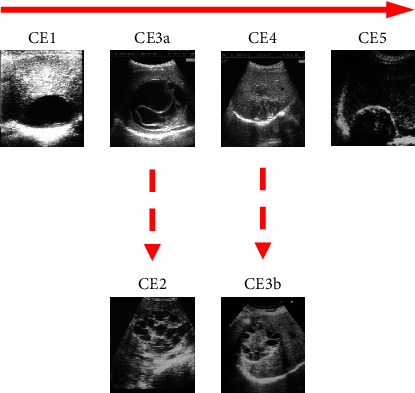
Natural history of CE liver cysts: active (CE1), transitional (CE3a), and inactive stages (CE4 and CE5). Reactivation can occur, giving rise to CE2 and CE3b cysts, from CE3a and CE4 stages, respectively.

In conclusion, the forced delay in surgical treatment of the lung cyst shows that the shift in cyst stage occurred during medical treatment from CE1 to CE3a, to CE4, occurs in the lung as well as in the liver and in other organs (Figure 3) [18, 19] and that entails the use of the more invasive pericystectomy instead of the endocystectomy performed when the cyst is CE1 or CE3a.

## Data Availability

Data are available upone request.
